# Supplementary material for: Examining the effectiveness of Prostatic hyperplasia education on the level of participant’s knowledge and awareness
Source: PLoS One. 2025 Jun 23;20(6):e0325653. doi: 10.1371/journal.pone.0325653 (PMC12184920; doi:10.1371/journal.pone.0325653)
Supplement: S1 File — (PDF) [file pone.0325653.s001.pdf]

SUPPLEMENTARY MATERIALS

Examining the effectiveness of prostatic hyperplasia education on the level of participant's knowledge and awareness

Hari Krismanuel[1*], Purnamawati Tjhin[2]

[1] Department of Surgery, Faculty of Medicine, Universitas Trisakti

[2]Department of Anatomy, Faculty of Medicine, Universitas Trisakti

* **Corresponding author:**

Hari Krismanuel, Department of Surgery, Universitas Trisakti, Jakarta, Indonesia

Email: hari_krismanuel@trisakti.ac.id

https://orcid.org/**0009-0002-2615-2363**

Content 1

Table S1. Management Options for Prostatic Hyperplasia 2

Table S2. Anonymized Data of Participants: Age, Education, and Test Scores 3

Table S3. Output of Data Analysis 4

Appendix S1. Pretest and Posttest Questions 8

Table S1. Management Options for Prostatic Hyperplasia

IPSS Score	Severity	Management Option
0-7	Mild	**Watchful waiting** and **lifestyle modification.**
		Lifestyle modification:
		- Limit fluid intake before bedtime or travel
		- Limit mild diuretics (e.g., caffeine, alcohol)
		- Limit bladder irritants (e.g., highly spiced or irritating foods)
		- Avoid constipation
		- Increase physical activity, including regular intense exercise
		- Weight management
		If **LUTS symptoms become bothersome**, pharmacotherapy or medical treatment can be considered
8-19	Moderate	**Pharmacotherapy/ Medical management.**
		If **pharmacological treatment fails** to alleviate symptoms, surgical intervention may be considered
20-35	Severe	**Surgical intervention or minimally invasive procedures**

Table S2. Anonymized Data of Participants: Age, Education, and Test Scores

Number	Participants	Age	Age Group	Education	Pre Test	Post Test
1	1	60_65	1	Elementary School	85.00	95.00
2	2	66_70	2	Elementary School	70.00	90.00
3	3	>70	3	Elementary School	55.00	90.00
4	4	60_65	1	Elementary School	90.00	65.00
5	5	66_70	2	Elementary School	75.00	60.00
6	6	>70	3	Elementary School	50.00	70.00
7	7	>70	3	Elementary School	60.00	60.00
8	8	>70	3	Elementary School	75.00	85.00
9	9	66_70	2	Elementary School	70.00	85.00
10	10	66_70	2	Elementary School	65.00	70.00
11	11	60_65	1	Elementary School	55.00	60.00
12	12	60_65	1	Elementary School	80.00	85.00
13	13	66_70	2	Elementary School	65.00	95.00
14	14	>70	3	Elementary School	70.00	80.00
15	15	66_70	2	Elementary School	75.00	75.00
16	16	60_65	1	Elementary School	75.00	75.00
17	17	>70	3	Elementary School	60.00	75.00
18	18	>70	3	Elementary School	70.00	80.00
19	19	60_65	1	Elementary School	55.00	80.00
20	20	66_70	2	Elementary School	65.00	70.00
21	21	60_65	1	Elementary School	65.00	80.00
22	22	>70	3	Elementary School	50.00	80.00
23	23	60_65	1	Elementary School	65.00	75.00
24	24	66_70	2	Elementary School	55.00	70.00
25	25	60_65	1	Elementary School	80.00	60.00
26	26	60_65	1	Elementary School	90.00	75.00
27	27	66_70	2	Elementary School	60.00	70.00
28	28	60_65	1	Elementary School	75.00	75.00
29	29	>70	3	Elementary School	50.00	60.00
30	30	>70	3	Elementary School	60.00	75.00
31	31	60_65	1	Elementary School	55.00	75.00
32	32	60_65	1	Elementary School	50.00	65.00

Table S3. Output of Data Analysis

Regression

Variables Entered/Removed[a]

Model	Variables Entered	Variables Removed	Method
1	pretest[b]	.	Enter

a. Dependent Variable: posttest

b. All requested variables entered.

Model Summary[b]

Model	R	R Square	Adjusted R Square	Std. Error of the Estimate
1	.186[a]	.034	.002	10.02738

a. Predictors: (Constant), pretest

b. Dependent Variable: posttest

ANOVA[a]

Model		Sum of Squares	df	Mean Square	F	Sig.
1	Regression	107.765	1	107.765	1.072	.309[b]
	Residual	3016.453	30	100.548		
	Total	3124.219	31			

a. Dependent Variable: posttest

b. Predictors: (Constant), pretest

Coefficients[a]

Model		Unstandardized Coefficients		Standardized Coefficients	t	Sig.
		B	Std. Error	Beta		
1	(Constant)	64.480	10.463		6.162	.000
	pretest	.161	.156	.186	1.035	.309

a. Dependent Variable: posttest

Residuals Statistics[a]

	Minimum	Maximum	Mean	Std. Deviation	N
Predicted Value	72.5377	78.9834	75.1562	1.86449	32
Residual	-17.37199	20.04518	.00000	9.86433	32
Std. Predicted Value	-1.404	2.053	.000	1.000	32
Std. Residual	-1.732	1.999	.000	.984	32

a. Dependent Variable: posttest

```
NPAR TESTS
  /K-S(NORMAL)=pretest posttest
  /MISSING ANALYSIS.
```

NPar Tests

One-Sample Kolmogorov-Smirnov Test

		pretest	posttest
N		32	32
Normal Parameters[a,b]	Mean	66.2500	75.1563
	Std. Deviation	11.57026	10.03899
Most Extreme Differences	Absolute	.116	.131
	Positive	.116	.131
	Negative	-.088	-.119
Test Statistic		.116	.131
Asymp. Sig. (2-tailed)		.200[c,d]	.174[c]

a. Test distribution is Normal.

b. Calculated from data.

c. Lilliefors Significance Correction.

d. This is a lower bound of the true significance.

```
*Nonparametric Tests: One Sample.
NPTESTS
  /ONESAMPLE TEST (RES_1)
  /MISSING SCOPE=ANALYSIS USERMISSING=EXCLUDE
  /CRITERIA ALPHA=0.05 CILEVEL=95.
```

Nonparametric Tests

Hypothesis Test Summary

	Null Hypothesis	Test	Sig.	Decision
1	The distribution of Unstandardized Residual is normal with mean ,00000 and standard deviation 9,86432692.	One-Sample Kolmogorov-Smirnov Test	.200[a,b]	Retain the null hypothesis.

Asymptotic significances are displayed. The significance level is ,050.

a. Lilliefors Corrected

b. This is a lower bound of the true significance.

One-Sample Kolmogorov-Smirnov Normal Test

Unstandardized Residual

One-Sample Kolmogorov-Smirnov Normal Test Summary

Total N		32
Most Extreme Differences	Absolute	.089
	Positive	.086
	Negative	-.089
Test Statistic		.089
Asymptotic Sig.(2-sided test)		.200[a,b]

a. Lilliefors Corrected

b. This is a lower bound of the true significance.

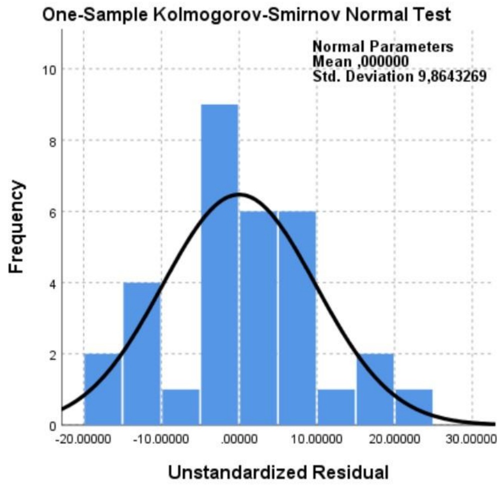


```
NPAR TESTS
  /K-S(NORMAL)=RES_1
  /MISSING ANALYSIS.
```

NPar Tests

One-Sample Kolmogorov-Smirnov Test

		Unstandardized Residual
N		32
Normal Parameters[a,b]	Mean	.0000000
	Std. Deviation	9.86432692
Most Extreme Differences	Absolute	.089
	Positive	.086
	Negative	-.089
Test Statistic		.089
Asymp. Sig. (2-tailed)		.200[c,d]

a. Test distribution is Normal.

b. Calculated from data.

c. Lilliefors Significance Correction.

d. This is a lower bound of the true significance.

```
T-TEST PAIRS=pretest WITH posttest (PAIRED)
  /CRITERIA=CI(.9500)
  /MISSING=ANALYSIS.
```

```
T-TEST PAIRS=pretest WITH posttest (PAIRED)
  /CRITERIA=CI(.9500)
  /MISSING=ANALYSIS.
```

T-Test

Paired Samples Statistics

		Mean	N	Std. Deviation	Std. Error Mean
Pair 1	pretest	66.2500	32	11.57026	2.04535
	posttest	75.1563	32	10.03899	1.77466

Paired Samples Correlations

		N	Correlation	Sig.
Pair 1	pretest & posttest	32	.186	.309

Paired Samples Test

		Paired Differences					t	df	Sig. (2-tailed)
					95% Confidence Interval of the Difference				
		Mean	Std. Deviation	Std. Error Mean	Lower	Upper			
Pair 1	pretest - posttest	-8.90625	13.83861	2.44634	-13.89560	-3.91690	-3.641	31	.001

```
NPAR TESTS
  /CHISQUARE=Agegroup
  /EXPECTED=EQUAL
  /MISSING ANALYSIS.
```

NPar Tests

Chi-Square Test

Frequencies

Agegroup

	Observed N	Expected N	Residual
1	13	10.7	2.3
2	9	10.7	-1.7
3	10	10.7	-.7
Total	32		

Test Statistics

	Agegroup
Chi-Square	.813[a]
df	2
Asymp. Sig.	.666

a. 0 cells (,0%) have expected frequencies less than 5. The minimum expected cell frequency is 10,7.

```
REGRESSION
  /DESCRIPTIVES MEAN STDDEV CORR SIG N
  /MISSING LISTWISE
  /STATISTICS COEFF OUTS CI(95) R ANOVA CHANGE
  /CRITERIA=PIN(.05) POUT(.10)
  /NOORIGIN
  /DEPENDENT Posttest
  /METHOD=ENTER Agegroup Pretest
  /SCATTERPLOT=(*ZPRED ,*ZRESID)
  /RESIDUALS HISTOGRAM(ZRESID) NORMPROB(ZRESID).
```

Regression

Descriptive Statistics

	Mean	Std. Deviation	N
Posttest	75.1563	10.03899	32
Agegroup	1.91	.856	32
Pretest	66.2500	11.57026	32

Correlations

		Posttest	Agegroup	Pretest
Pearson Correlation	Posttest	1.000	.058	.186
	Agegroup	.058	1.000	-.395
	Pretest	.186	-.395	1.000
Sig. (1-tailed)	Posttest	.	.376	.154
	Agegroup	.376	.	.013
	Pretest	.154	.013	.
N	Posttest	32	32	32
	Agegroup	32	32	32
	Pretest	32	32	32

Variables Entered/Removed[a]

Model	Variables Entered	Variables Removed	Method
1	Pretest, Agegroup[b]	.	Enter

a. Dependent Variable: Posttest

b. All requested variables entered.

Model Summary[b]

Model	R	R Square	Adjusted R Square	Std. Error of the Estimate	Change Statistics				
					R Square Change	F Change	df1	df2	Sig. F Change
1	.234[a]	.055	-.010	10.09019	.055	.843	2	29	.441

a. Predictors: (Constant), Pretest, Agegroup

b. Dependent Variable: Posttest

ANOVA[a]

Model		Sum of Squares	df	Mean Square	F	Sig.
1	Regression	171.674	2	85.837	.843	.441[b]
	Residual	2952.545	29	101.812		
	Total	3124.219	31			

a. Dependent Variable: Posttest

b. Predictors: (Constant), Pretest, Agegroup

Coefficients[a]

Model		Unstandardized Coefficients		Standardized Coefficients	t	Sig.	95,0% Confidence Interval for B	
		B	Std. Error	Beta			Lower Bound	Upper Bound
1	(Constant)	57.467	13.756		4.178	.000	29.333	85.601
	Agegroup	1.826	2.304	.156	.792	.435	-2.887	6.538
	Pretest	.214	.170	.247	1.258	.218	-.134	.563

a. Dependent Variable: Posttest

Residuals Statistics[a]

	Minimum	Maximum	Mean	Std. Deviation	N
Predicted Value	70.0165	79.0297	75.1562	2.35327	32
Residual	-17.20411	19.94070	.00000	9.75927	32
Std. Predicted Value	-2.184	1.646	.000	1.000	32
Std. Residual	-1.705	1.976	.000	.967	32

a. Dependent Variable: Posttest

Appendix S1. Pretest and Posttest Questions

SOAL PRETEST

Pilihlah jawaban B bila benar dan S bila salah dengan memberi tanda silang pada jawaban yang dipilih.

1. Kelenjar prostat hanya dimiliki oleh para pria. B S
2. Hiperplasia prostat adalah pembesaran kelenjar prostat jinak. B S
3. Keadaan ini dialami oleh para pria, terutama pada usia ≥ 60 tahun B S
4. Angka kejadian Hiperplasia Prostat pada pria berusia 51 – 60 th sekitar 50 %. B S
5. Hiperplasia Prostat menyebabkan aliran urine menjadi tidak lancar dan buang air kecil terasa tidak tuntas. B S
6. Kumpulan gejala yang terjadi akibat adanya Hiperplasia Prostat disebut sindroma LUTS. B S
7. International Prostate Symptom Score (I-PSS) adalah system penilaian berat ringannya sindroma LUTS. B S
8. Sindroma LUTS dibagi menjadi sindroma obstruktif dan iritatif. B S
9. Sindroma LUTS dikatakan berat bila skornya 8 – 19. B S
10. Sindroma LUTS dikatakan sedang bila skornya 1 – 7. B S
11. Salah satu gejala HP adalah BAK tidak tuntas/ tidak lampias. B S
12. Nocturia adalah gejala sering buang air kecil di siang hari. B S
13. Straining adalah gejala sering kencing pada penderita Hiperplasia Prostat. B S
14. Urgency adalah perasaan sulit menahan buang air kecil. B S
15. Weak stream adalah pancaran kencing lemah. B S
16. Frequency adalah sering buang air kecil di malam hari. B S
17. Intermittency adalah buang air kecil berhenti, kemudian mulai lagi saat miksi. B S
18. Selain menilainya berat ringannya sindroma LUTS, I-PSS juga menilai kualitas hidup seseorang akibat adanya sindroma LUTS. B S
19. Retensi urin adalah salah satu tanda adanya Hiperplasia Prostat yang berat. B S
20. Colok Dubur adalah pemeriksaan klinis untuk menilai HiperplasiaB Prostat. S

Translation

PRETEST QUESTIONS

Select **B** if the statement is **true** and **S** if the statement is **false** by marking the chosen answer.

1. The prostate gland is only found in men. **B S**
2. Benign prostatic hyperplasia (BPH) is the enlargement of the prostate gland. **B S**
3. This condition occurs in men, especially those aged ≥ 60 years. **B S**
4. The incidence of Benign Prostatic Hyperplasia in men aged 51–60 years is approximately 50%. **B S**
5. Benign Prostatic Hyperplasia causes urinary flow to become obstructed and results in incomplete bladder emptying. **B S**
6. The collection of symptoms caused by Benign Prostatic Hyperplasia is called the LUTS syndrome. **B S**
7. The International Prostate Symptom Score (I-PSS) is a system for assessing the severity of LUTS. **B S**
8. LUTS syndrome is divided into obstructive and irritative syndromes. **B S**
9. LUTS is classified as severe when the score is between 8 and 19. **B S**
10. LUTS is classified as moderate when the score is between 1 and 7. **B S**
11. One symptom of BPH is incomplete bladder emptying. **B S**
12. Nocturia is the symptom of frequent urination at night. **B S**
13. Straining is a symptom of difficulty urinating in patients with Benign Prostatic Hyperplasia. **B S**
14. Urgency is the feeling of difficulty holding urine. **B S**
15. Weak stream refers to a weak urine flow. **B S**
16. Frequency refers to frequent urination at night. **B S**
17. Intermittency refers to urine flow stopping and then resuming during urination. **B S**
18. Besides assessing the severity of LUTS, I-PSS also evaluates the quality of life of individuals with LUTS. **B S**
19. Urinary retention is one of the signs of severe Benign Prostatic Hyperplasia. **B S**
20. Digital rectal examination is a clinical test used to assess Benign Prostatic Hyperplasia.

    B S

SOAL POST TEST

Pilihlah jawaban B bila benar dan S bila salah dengan memberi tanda silang pada jawaban yang dipilih.

1. Kelenjar prostat hanya dimiliki oleh para pria. B S
2. Hiperplasia prostat adalah pembesaran kelenjar prostat jinak. B S
3. Keadaan ini dialami oleh para pria, terutama pada usia $\geq$ 60 tahun B S
4. Angka kejadian Hiperplasia Prostat pada pria berusia 51 – 60 th sekitar 50 %. B S
5. Hiperplasia Prostat menyebabkan aliran urine menjadi tidak lancar dan buang air kecil terasa tidak tuntas. B S
6. Kumpulan gejala yang terjadi akibat adanya Hiperplasia Prostat disebut sindroma LUTS. B S
7. International Prostate Symptom Score (I-PSS) adalah system penilaian berat ringannya sindroma LUTS. B S
8. Sindroma LUTS dibagi menjadi sindroma obstruktif dan iritatif. B S
9. Sindroma LUTS dikatakan berat bila skornya 8 – 19. B S
10. Sindroma LUTS dikatakan sedang bila skornya 1 – 7. B S
11. Salah satu gejala HP adalah BAK tidak tuntas/ tidak lampias. B S
12. Nocturia adalah gejala sering buang air kecil di siang hari. B S
13. Straining adalah gejala sering kencing pada penderita Hiperplasia Prostat. B S
14. Urgency adalah perasaan sulit menahan buang air kecil. B S
15. Weak stream adalah pancaran kencing lemah. B S
16. Frequency adalah sering buang air kecil di malam hari. B S
17. Intermittency adalah buang air kecil berhenti, kemudian mulai lagi saat miksi. B S
18. Selain menilainya berat ringannya sindroma LUTS, I-PSS juga menilai kualitas hidup seseorang akibat adanya sindroma LUTS. B S
19. Retensi urin adalah salah satu tanda adanya Hiperplasia Prostat yang berat. B S
20. Colok Dubur adalah pemeriksaan klinis untuk menilai Hiperplasia Prostat. B S

Translation

POSTEST QUESTIONS

Select **B** if the statement is **true** and **S** if the statement is **false** by marking the chosen answer.

1. The prostate gland is only found in men. **B S**
2. Benign prostatic hyperplasia (BPH) is the enlargement of the prostate gland. **B S**
3. This condition occurs in men, especially those aged ≥ 60 years. **B S**
4. The incidence of Benign Prostatic Hyperplasia in men aged 51–60 years is approximately 50%. **B S**
5. Benign Prostatic Hyperplasia causes urinary flow to become obstructed and results in incomplete bladder emptying. **B S**
6. The collection of symptoms caused by Benign Prostatic Hyperplasia is called the LUTS syndrome. **B S**
7. The International Prostate Symptom Score (I-PSS) is a system for assessing the severity of LUTS. **B S**
8. LUTS syndrome is divided into obstructive and irritative syndromes. **B S**
9. LUTS is classified as severe when the score is between 8 and 19. **B S**
10. LUTS is classified as moderate when the score is between 1 and 7. **B S**
11. One symptom of BPH is incomplete bladder emptying. **B S**
12. Nocturia is the symptom of frequent urination at night. **B S**
13. Straining is a symptom of difficulty urinating in patients with Benign Prostatic Hyperplasia. **B S**
14. Urgency is the feeling of difficulty holding urine. **B S**
15. Weak stream refers to a weak urine flow. **B S**
16. Frequency refers to frequent urination at night. **B S**
17. Intermittency refers to urine flow stopping and then resuming during urination. **B S**
18. Besides assessing the severity of LUTS, I-PSS also evaluates the quality of life of individuals with LUTS. **B S**
19. Urinary retention is one of the signs of severe Benign Prostatic Hyperplasia. **B S**
20. Digital rectal examination is a clinical test used to assess Benign Prostatic Hyperplasia.

    B S
