# Supplementary material for: Examining the effectiveness of Prostatic hyperplasia education on the level of participant’s knowledge and awareness
Source: PLoS One. 2025 Jun 23;20(6):e0325653. doi: 10.1371/journal.pone.0325653 (PMC12184920; doi:10.1371/journal.pone.0325653)
Supplement: Attachment — Submitted filename: RESPONSE TO REVIEWERS.pdf [file pone.0325653.s003.pdf]

RESPONSE TO REVIEWERS

1. Is the manuscript technically sound, and does the data support the conclusions?

The manuscript must describe a technically sound piece of scientific research with data that supports the conclusions. Experiments must have been conducted rigorously, with appropriate controls, replication, and sample sizes. The conclusions must be drawn appropriately based on the data presented.

Reviewer #1: Yes

Reviewer #2: Partly

Reviewer #3: Yes

Reviewer #4: Yes

RESPONSE TO REVIEWERS

Dear Reviewers,

We sincerely appreciate your thorough review of our manuscript titled **"Examining the Effectiveness of Prostatic Hyperplasia Education on the Level of Participants' Knowledge and Awareness."** We are grateful for your constructive feedback, which has helped us refine and improve the clarity and rigor of our study.

Regarding the question posed by the editor on whether the manuscript is technically sound and whether the data support our conclusions, we note that three reviewers (Reviewers #1, #3, and #4) responded affirmatively, while Reviewer #2 indicated "Partly." However, no specific concerns were provided regarding which aspect of the study was deemed partial in technical soundness.

To address the concerns raised by Reviewer #2, we have made the following clarifications and improvements:

Response to Reviewer #2:

Thank you for your valuable feedback. We appreciate your insights and have carefully addressed your concerns regarding the technical soundness of the manuscript and the adequacy of the data in supporting our conclusions.

1. **Study Design and Rationale**

   Our study employs a well-established quasi-experimental pretest-posttest design without a control group, which is appropriate for evaluating educational interventions. We are confident that our study meets the technical rigor required for evaluating educational interventions, and that the data presented provide strong support for our

conclusions. While a control group could have provided additional comparison, our focus was to assess within-group knowledge improvement directly attributable to the intervention.

2. **Statistical Analysis and Transparency**
The statistical analysis was conducted independently by the authors using SPSS, applying appropriate methods such as paired t-tests and effect size calculations (Cohen's d) to comprehensively assess the intervention's impact. We explicitly described this in the Methods section and presented the detailed statistical results in the Results section to ensure full transparency.

3. **Clarification on the Pretest-Posttest Questionnaire**
The questionnaire was adapted from the validated International Prostate Symptom Score (I-PSS) tool. We have now included a clearer explanation of its components and the rationale for its use in the revised manuscript.

4. **Balance Between Statistical analysis, Quantitative and Qualitative Findings**
While statistical analysis was essential in demonstrating the intervention's effectiveness, we also presented qualitative findings that provided deeper insights into participants' understanding and perceptions. These qualitative results are now emphasized in both the Results and Discussion sections to ensure a balanced perspective.

We believe these revisions further strengthen the manuscript's clarity and rigor. If Reviewer #2 has specific concerns beyond these points, we would greatly appreciate further clarification.

Best regards,

[Dr. Hari Krismanuel]
[Universitas Trisakti]

2. Has the statistical analysis been performed appropriately and rigorously?

Reviewer #1: Yes

Reviewer #2: Yes

Reviewer #3: Yes

Reviewer #4: Yes

RESPONSE TO REVIEWERS

Dear Reviewers,

We sincerely appreciate the reviewers' positive evaluations of our statistical analysis. As all four reviewers (Reviewers #1, #2, #3, and #4) have confirmed that the statistical analysis was

performed appropriately and rigorously, we have maintained our analytical approach in the revised manuscript.

Nonetheless, we have carefully reviewed the statistical methods to ensure clarity and have provided additional explanations where necessary to enhance the transparency of our analysis. We appreciate the reviewers' recognition of the robustness of our statistical approach and thank them for their valuable insights.

Best regards,

[Dr. Hari Krismanuel]
[Universitas Trisakti]

3. Have the authors made all data underlying the findings in their manuscript fully available?

The PLOS Data policy requires authors to make all data underlying the findings described in their manuscript fully available without restriction, with rare exceptions (please refer to the Data Availability Statement in the manuscript PDF file). The data should be provided as part of the manuscript or its supporting information, or deposited to a public repository. For example, in addition to summary statistics, the data points behind means, medians, and variance measures should be available. If there are restrictions on publicly sharing data—e.g. participant privacy or use of data from a third party—those must be specified.

Reviewer #1: Yes

Reviewer #2: No

Reviewer #3: Yes

Reviewer #4: Yes

RESPONSE TO REVIEWERS

Dear Reviewers,

We sincerely appreciate your comments regarding data availability. While three reviewers (Reviewers #1, #3, and #4) confirmed that the data have been made fully available, we acknowledge the concern raised by Reviewer #2.

To clarify, we fully support data transparency while adhering to **ethical guidelines and participant privacy protection**. The original dataset contains sensitive personal information, including participant names and addresses, which must remain confidential. However, in response to these concerns, we have taken the following steps:

- **Request for Clarification:** We respectfully request Reviewer 2 to provide further clarification regarding the reasons behind their "No" response. This will enable us to

better address their specific concerns and ensure that we have provided all necessary data in an appropriate format.

- **Anonymization of data**: We have prepared a version of the dataset where participant names are represented only by initials, and all address information has been removed.
- We have added the **Data Availability Statement** section to explicitly mention the anonymization process and to clarify that anonymized participant data are securely stored and available as Supplemental Materials through [repository link]."
- **To further enhance transparency and address the reviewer's concern about the availability of the questionnaire and detailed results,** we will include the complete pre-test and post-test questionnaire, the detailed SPSS output of our statistical analysis, and a brief summary of the management options discussed during the educational session as Supplemental Materials. This will provide a more comprehensive understanding of the assessment tools and the detailed findings of our study.

We believe these measures address Reviewer #2's concerns while maintaining the integrity and transparency of our research.

Best regards,

[Dr. Hari Krismanuel]
[Universitas Trisakti]

4. Is the manuscript presented in an intelligible fashion and written in standard English?

PLOS ONE does not copyedit accepted manuscripts, so the language in submitted articles must be clear, correct, and unambiguous. Any typographical or grammatical errors should be corrected at revision, so please note any specific errors here.

Reviewer #1: Yes

Reviewer #2: Yes

Reviewer #3: Yes

Reviewer #4: Yes

RESPONSE TO REVIEWERS

Dear Reviewers,

We sincerely appreciate your feedback on the clarity and readability of our manuscript. We are pleased to note that all reviewers have confirmed that the manuscript is presented in an intelligible fashion and written in standard English.

Thank you for your time and valuable insights.

Best regards,

[Dr. Hari Krismanuel]
[Universitas Trisakti]

5. Review Comments to the Author

Please use the space provided to explain your answers to the questions above. You may also include additional comments for the author, including concerns about dual publication, research ethics, or publication ethics. (Please upload your review as an attachment if it exceeds 20,000 characters)

Reviewer #1: The manuscript presents a community-based educational intervention aimed at improving knowledge and awareness about Prostatic Hyperplasia (PH) among elderly men in Bogor, Indonesia. Overall, the study contributes meaningfully to the field, especially in addressing health education gaps for elderly populations in low-resource settings. Below is the detailed feedback regarding the manuscript:
Technical Soundness and Data Support: The manuscript describes a well-structured quasi-experimental study with a pretest-posttest design. The statistical analysis, including paired t-tests and Cohen's d calculation, is appropriately applied to assess the intervention's effectiveness. The quantitative results are compelling, with a significant increase in knowledge scores post-intervention. Qualitative data enrich the findings by providing deeper insights into participant perceptions. However, the lack of a control group limits the ability to establish causality. Future iterations could consider incorporating control groups to strengthen the validity of the conclusions.
Statistical Analysis: The statistical methods employed, including normality tests and effect size calculations, are rigorous and align with the study's objectives. The authors have adequately described the steps taken to ensure the robustness of the analysis. The effect size (Cohen's d = 0.82) indicates a large practical impact of the intervention, which is encouraging.
Data Availability: The data availability statement is adequate, and all relevant data are included within the manuscript and its supporting files. However, it would be beneficial for the authors to specify whether the raw dataset (e.g., anonymized pretest and posttest scores) is available in a public repository for reproducibility.
Language and Presentation: The manuscript is written in clear and standard English, making it accessible to a wide audience. The structure of the paper is logical, and the arguments are easy to follow. While there are no major grammatical errors, minor typographical errors should be addressed during revision.
Strengths of the Study: The integration of the International Prostatic Symptom Score (I-PSS) into a community education setting is novel and provides a practical tool for participants to

self-assess their symptoms. The mixed-methods approach adds depth to the findings by combining quantitative results with qualitative insights. The scalability and low-cost nature of the intervention make it suitable for broader applications in similar settings.
Limitations and Suggestions for Improvement: The lack of a control group is a significant limitation. Future studies should aim to include a control group to strengthen causal inferences. The sample size, while adequate for initial findings, could be expanded to improve generalizability. Including family members in the educational sessions may enhance the program's impact and encourage broader awareness. More interactive elements, such as case studies or role-playing, could further engage participants and reinforce learning.
Ethics and Reporting Standards: The study adheres to ethical standards, with appropriate approval obtained and clear documentation of informed consent procedures. The manuscript follows reporting guidelines and includes sufficient methodological details to ensure reproducibility.
Conclusion: The study provides strong evidence for the effectiveness of community-based education using the I-PSS tool. It demonstrates potential as a scalable, low-cost intervention to address health education gaps in low-resource settings. The authors have made a valuable contribution to the field of community health education.

Reviewer #2: The article is an interesting one but lacks a few basic components, like the purpose of the study is not clear. The article is about educational intervention and patient education but the maximum emphasis is on the statistical details. The pre-test/post-test questionnaire is not provided nor discussed. The results just mention the difference between cumulative scores without details of components (like symptoms, management options, etc).

Statistical details may be reviewed by a statistician

Reviewer #3: The data regarding the education level of participants should be added to this study to identify the relation between the education level and the knowledge of prostate hyperplasia. Therefore, the conclusion should also mention the effectiveness of education level and the impact of health education in the community regarding prostate hyperplasia

Reviewer #4: This study can be applied in a daily urology clinical setting. The data used in this research is reliable and has been handled appropriately. The next research about early screening and treatment of benign prostatic hyperplasia can be developed from these studies, by taking larger samples or populations.

Response to Reviewers

Dear Reviewers,

We sincerely appreciate the constructive feedback on our manuscript. Below, we address each comment in detail and describe the revisions we have made to enhance the clarity and rigor of our study.

RESPONSE TO REVIEWER #1

Dear Reviewer #1,

We sincerely appreciate your thorough and constructive review of our manuscript. Your comments have been very helpful in improving the clarity and rigor of our study. Below are our detailed responses to your feedback:

1. **Technical Soundness and Data Support**

   Thank you for acknowledging the structured approach of our quasi-experimental study and the robustness of our statistical analysis. Regarding your concern about the lack of a control group, we acknowledge that having a control group would strengthen causal inferences. However, our study was designed as a one-group pretest-posttest study due to practical and ethical considerations. Since the primary goal of this research was to evaluate the effectiveness of an educational intervention in improving knowledge and awareness of Prostatic Hyperplasia (PH) among elderly men, we focused on measuring individual changes before and after the intervention within the same group of participants. This approach allowed us to assess the direct impact of the education without withholding valuable health information from any participants, which would have been required in a controlled setting.

   Additionally, the statistical significance of our findings (Cohen's d = 0.82) indicates a strong effect size, supporting the effectiveness of the intervention. While a future study incorporating a control group would be ideal, we believe that our current approach still provides valuable insights, particularly in low-resource settings where access to healthcare education is limited.

2. **Statistical Analysis**

   We are grateful for your positive assessment of our statistical methods. We ensured that all analyses, including normality testing, paired t-tests, and effect size calculations, were conducted rigorously to ensure the reliability of our findings.

3. **Data Availability**

   We appreciate your suggestion regarding data sharing. In compliance with ethical guidelines and participant privacy protection, we have made anonymized versions of the pretest and posttest scores available. Identifiable information, such as participant names and addresses, has been omitted to prevent any potential breaches of confidentiality. We have also updated our Data Availability Statement to clarify this.

4. **Language and Presentation**

   Thank you for your kind words regarding the clarity of our manuscript. We have carefully reviewed the text for minor typographical errors and have revised them accordingly.

5. **Strengths of the Study**

We appreciate your recognition of the novelty and strengths of our study, particularly the integration of the International Prostatic Symptom Score (I-PSS) into a community education setting. This aspect was a key focus of our study, as it provides participants with a practical self-assessment tool to better understand their symptoms and potentially seek timely medical consultation. Additionally, we acknowledge the value of the mixed-methods approach in enhancing the depth of our findings by incorporating both quantitative and qualitative insights. The qualitative feedback from participants provided valuable context to the numerical data, offering a more comprehensive understanding of the intervention's effectiveness.

Furthermore, the scalability and cost-effectiveness of the intervention make it suitable for broader applications in similar community settings, particularly in resource-limited areas where access to specialized care may be restricted. By using a structured but adaptable educational framework, this program can be replicated or modified to address other health conditions. We have emphasized these strengths in the manuscript to highlight the significance of our approach and its potential for future implementation and improvement.

6. **Limitations and Suggestions for Improvement:**

Regarding the study limitations, we acknowledge the points you raised and have explicitly stated in the manuscript that future studies should consider incorporating a control group and expanding the sample size to enhance generalizability.

Regarding the inclusion of a control group, while it is beneficial for strengthening causal inferences, we opted for a **one-group pre-test and post-test design**, as it allows us to directly measure knowledge improvement within the same participants. Additionally, we implemented strict exclusion criteria, ensuring that participants who had previously received similar education were not included in the study. This step was taken to minimize bias in assessing the true impact of the intervention. However, we recognize that despite this measure, some inherent limitations remain in the absence of a control group.

One key limitation is the potential influence of external factors—such as prior exposure to related health information through media or personal discussions—which may contribute to knowledge improvement beyond the intervention itself. Another consideration is the **testing effect**, where taking a pre-test might increase participants' awareness of the topic, making them more receptive to learning. Additionally, **regression to the mean** may occur if participants with initially low scores naturally improve over time, independent of the intervention.

Furthermore, implementing a control group in a community-based educational setting poses several challenges. **Selection bias** may arise due to differences in motivation, health awareness, or baseline knowledge between intervention and control groups. **Contamination risk** is also a concern, as participants in the control group might indirectly receive information from those in the intervention group, thereby diluting the intended effect of the educational program. Moreover, there are **ethical considerations**, as withholding beneficial health education from a control group may not be justifiable. Finally, a **control group would require additional logistical and resource commitments**, which could limit feasibility in community settings with constrained funding and personnel.

We also considered the suggestion to involve **family members** in the educational program. While this could potentially enhance the intervention's impact, it may also introduce **greater variability in participant characteristics**, such as differences in

age, educational background, and gender. These factors could affect the homogeneity of the study population and introduce additional biases, making it more challenging to interpret the intervention's effectiveness accurately.

Regarding the incorporation of **more interactive elements**, our educational intervention already included PowerPoint presentations projected onto a screen, pre- and post-tests to assess knowledge improvement, and a **structured Q&A session** that allowed participants to engage actively with the material. Additionally, participants were asked about their perceptions of the educational content and their understanding of the material. While case studies or role-playing could be valuable additions, incorporating these elements would require additional time and resources, which may not have been feasible within the structure of our community-based educational program. Nonetheless, the current interactive components were well-received and provided meaningful engagement within the study's scope. Future studies could explore further enhancements in interactivity based on available resources and participant preferences.

7. **Ethics and Reporting Standards**

   We appreciate your positive feedback on the ethical standards and reporting of our study. We have ensured that all necessary ethical approvals and informed consent procedures are well-documented in the manuscript.

Once again, we sincerely appreciate your insightful comments, which have strengthened our manuscript. We hope that our revisions and clarifications address your concerns.

Best regards,

[Dr. Hari Krismanuel]
[Universitas Trisakti]

RESPONSE TO REVIEWER #2

Dear Reviewer #2,

Thank you for your constructive feedback on our manuscript. We appreciate your valuable insights and have carefully addressed your concerns as follows:

1. **Clarity of the Study Purpose**

   We appreciate the reviewer's concern regarding the study's purpose. The study aims to assess the effectiveness of community-based education on prostatic hyperplasia (PH) using the International Prostatic Symptom Score (I-PSS). This is clearly stated in the abstract and is reflected in our study design.

   This objective is aligned with the study design and methodology, which focus on assessing the impact of educational interventions on participants' knowledge and awareness. The use of I-PSS further underscores the structured approach in symptom self-assessment, reinforcing the study's purpose.

To ensure further clarity, we are open to refining the wording in the introduction or methods section if the reviewer suggests a specific area where additional explanation is needed. However, we believe that the study's aim has been well articulated within the abstract and throughout the manuscript.

2. **Emphasis on Statistical Details**

We appreciate your feedback regarding the balance between statistical details and the core educational aspects of our study. While statistical analysis is crucial in assessing the effectiveness of our intervention, we recognize the need to present the findings in a more intuitive and clinically relevant manner.

In response to your comment, we have refined the *Results* section to provide a clearer narrative that emphasizes the practical implications of the findings rather than focusing excessively on statistical intricacies. We have also expanded the discussion of the pre-test/post-test questionnaire to include details on the components assessed (e.g., symptoms recognition, and knowledge of management options) to offer a more comprehensive understanding of knowledge improvement.

Additionally, the *Discussion* section has been revised to ensure a balanced integration of statistical outcomes with their real-world significance. We have placed greater emphasis on how the intervention influenced participants' awareness, symptom recognition, and potential behavioral changes in seeking medical care. These revisions aim to enhance readability and accessibility for a broader audience, including clinicians and public health practitioners.

We hope these improvements address your concerns and strengthen the overall clarity and impact of the manuscript. Thank you for your valuable insights.

3 **Pretest-Posttest Questionnaire and Results**

We appreciate the reviewer's comments regarding the pretest and posttest assessments and the balance between statistical analysis, quantitative findings, and qualitative findings.
The **pretest and posttest questions** were based on the **International Prostatic Symptom Score (I-PSS)**, a validated tool for assessing **lower urinary tract symptoms (LUTS) related to Prostatic Hyperplasia (PH)**. This questionnaire was designed to evaluate participants' **knowledge of LUTS symptoms**, **rather than management or treatment options**, in alignment with the study's objective of enhancing awareness and encouraging early medical consultation.
We would like to clarify that while **management options were briefly mentioned during the educational session to provide participants with additional knowledge, they were not included in the pretest and posttest assessments.** The questionnaire was strictly designed to assess knowledge of LUTS symptoms based on the International Prostatic Symptom Score (I-PSS), in line with our study's objective of raising awareness about early symptom recognition and encouraging medical

consultation. We have now clarified this distinction in the manuscript to prevent any potential misunderstanding.

We acknowledge the reviewer's concern that the discussion may seem to emphasize statistical analysis. However, our study does not rely solely on **quantitative results**—we have also incorporated **qualitative findings** to provide a more comprehensive understanding of the intervention's impact. **While statistical analysis is essential for objectively assessing changes in participants' knowledge, qualitative data enriches these findings by capturing participants' perceptions and experiences in their own words.**

In the **Results section**, we reported a **statistically significant improvement** in participants' understanding of LUTS following the intervention. Our primary analysis focused on **overall knowledge change**, rather than breaking down each symptom component, as the study aimed to measure general awareness rather than symptom-specific differentiation.

To further support the quantitative findings, we included **qualitative analysis** from focus group discussions, which revealed two major themes:

1. **Increased Understanding of PH Symptoms** – Participants reported improved awareness of urinary symptoms and felt more confident in identifying them early.
2. **Empowerment Through the I-PSS Tool** – Many participants found the I-PSS form useful for self-assessment and felt more in control of their health.

The integration of **statistical, quantitative and qualitative findings** highlights the **effectiveness of the intervention** in improving participants' **knowledge and awareness** of PH. The **significant changes** in pretest and posttest scores, along with **positive qualitative feedback**, suggest that community-based educational programs using tools like I-PSS can have a meaningful impact on promoting **early detection and proactive health-seeking behavior** among elderly populations.

To **clarify these points**, we have now explicitly stated these aspects in the **Methods, Results, and Discussion** sections. We believe that incorporating **statistical, quantitative findings, and qualitative evidence** provides a well-rounded view of the intervention's impact, addressing the reviewer's concerns regarding balance in the discussion.

To enhance transparency and provide a more comprehensive understanding of our assessment tools and results, we have now included the following in the Supplemental Materials:

- **Table S1**: A brief summary of the management options discussed during the educational session
- **Table S2**: Anonymized participant data
- **Table S3**: The detailed SPSS output of our statistical analysis
- **Appendix S1**: The complete pre-test and post-test questionnaire

We confirm that all participant data presented in Table S2 have been fully anonymized to ensure confidentiality and comply with ethical research standards.

These additions ensure transparency and provide a clearer insight into our methodology and findings.

4 **Statistical Analysis Review**

We appreciate the reviewer's acknowledgment of the accuracy and rigor of the statistical analysis. The statistical analysis, including paired t-tests for pretest-posttest comparisons and effect size calculations (Cohen's d), was conducted independently by the author using SPSS. To ensure transparency, we have explicitly described the statistical methods in the Methods section and provided the corresponding results in the Results section. The detailed presentation of statistical findings serves as evidence that the analyses were performed by ourselves, without the involvement of an external statistician. **Furthermore, we have the complete output from the statistical analyses conducted in SPSS, which includes Kolmogorov-Smirnov Test, Paired Samples T-Test, ANOVA tables, regression coefficients, residual plots, and other relevant statistics. This comprehensive output serves as additional evidence that the analyses were performed independently by the author. The complete dataset and statistical output are available in the Supplemental Materials.**

We appreciate your insightful comments, which have helped refine our manuscript. We hope that our revisions adequately address your concerns and improve the clarity of our study.

Best regards,

[Dr. Hari Krismanuel]
[Universitas Trisakti]

RESPONSE TO REVIEWER #3

Dear Reviewer #3,

Thank you for your insightful comments and suggestions. We appreciate your recommendation to include education level in the analysis and discussion. Below, we provide our response to your concerns:

1. **Education Level of Participants**

   We acknowledge the importance of considering education level in health education studies. In our study, all participants were elderly men (≥ 60 years) from rural areas with a similar educational background (elementary school level). This **homogeneity in educational background** minimizes variability across age groups and effectively controls for potential confounders related to differences in education level and knowledge improvement.

Given this homogeneity, we did not conduct a subgroup analysis to examine the relationship between education level and knowledge improvement. Instead, our focus was on assessing the overall effectiveness of the educational intervention for this specific demographic group.

2. **Clarification on Study Purpose**

   The primary objective of this study was to evaluate the effectiveness of a community-based education program on prostatic hyperplasia (PH) in enhancing knowledge and awareness among elderly male participants, who are a high-risk group for this condition. The study was not designed to explore the relationship between education level and knowledge gain but rather to assess whether structured health education could effectively enhance awareness and prompt early health-seeking behavior in this population. Unlike prior studies conducted in clinical settings, this research focused on a single-group pretest-posttest design to assess overall improvement in knowledge rather than comparing knowledge gains across different education levels. Given the uniformity in participants' education levels, analyzing its impact on knowledge acquisition would not yield meaningful comparative insights.

3. **Demographic Data Inclusion**

   While we did not emphasize education level as a variable influencing the study outcome, we recognize its relevance in providing context. To address this, we have included participant education level in the Demographic Table in the Methods section. Additionally, we have added a brief discussion on how uniformity in education level helps in controlling potential confounders related to knowledge differences in the Discussion section. Furthermore, we have revised the Conclusion to clarify that due to the relatively homogeneous educational background of participants, the influence of education level on the effectiveness of the intervention could not be analyzed. Therefore, our findings focus on the overall knowledge improvement observed before and after the educational intervention without comparing its impact based on education level.

We appreciate your valuable feedback and believe that these revisions strengthen the clarity and contribution of our study. We hope that our response adequately addresses your concerns.

Best regards,

[Dr. Hari Krismanuel]
[Universitas Trisakti]

RESPONSE TO REVIEWER #4

Dear Reviewer #4,

Clinical relevance and future research directions:
We appreciate the recognition that our study has practical applications in **urology clinical settings**. We agree that **future research could explore early screening and treatment strategies for BPH**, with **larger sample sizes and expanded populations**, and have mentioned this in the **Discussion** section.

Best regards,

[Dr. Hari Krismanuel]
[Universitas Trisakti]

Final Remarks

We are grateful for the reviewers' insights, which have significantly strengthened our manuscript. We have carefully addressed all comments and revised the manuscript accordingly.

Thank you for your time and consideration. We look forward to your feedback.

Best regards,

[Dr. Hari Krismanuel]
[Universitas Trisakti]
